# The use of episodic future thinking in people with overweight or obesity: A scoping review

**DOI:** 10.1097/MD.0000000000034269

**Published:** 2023-07-28

**Authors:** Yuchen Liu, Sufang Huang, Danni Feng, Xiaorong Lang, Quan Wang, Kexin Zhang

**Affiliations:** a Tongji Hospital, Tongji Medical College, Huazhong University of Science and Technology, Jiefang Avenue 1095#, Wuhan 430030, Hubei, P.R.China; b School of Nursing, Tongji Medical College, Huazhong University of Science and Technology, Wuhan, Hubei, P.R. China.

**Keywords:** delay discounting, episodic future thinking, food choice, intervention, obesity, overweight, scoping review

## Abstract

A growing number of studies have applied Episodic Future Thinking (EFT) to cognitive interventions in specific population. However, The variability in study populations may lead to inconsistent results and present challenges in the optimal intervention approach and scope of adaptation. This scoping review aimed to identify and describe specific methods, considerations, and results collected and reported in randomized controlled trials of EFT applied to diet and weight management in people with overweight or obesity. A systematic scoping review was conducted by published guidelines for this review. We conducted a structured search of English-language articles in Web of Science, PubMed, Embase, CINAHL, ProQuest, and Cochrane, with the literature focusing on studies published up to December 28, 2022. After screening and full-text review, 16 studies were included. The studies included people of all ages with overweight or obesity, including women, children, and home-based EFT interventions. The vast majority of intervention studies screened participants for psychological characteristics, and the effects of EFT applied in people with overweight or obesity were somewhat significant, although there was some variation in the literature. Although the individual heterogeneity of studies makes the synthesis of results somewhat variable, it still demonstrates the breadth and accessibility of EFT interventions in people with overweight or obesity. The application of EFT to individualized interventions in people with overweight or obesity is a further complement and optimization of weight management through behavioral cognitive therapy.

## 1. Introduction

The global obesity rate has almost tripled since 1975, reaching over 650 million.^[1]^ The global epidemic of overweight or obesity is becoming a serious public health challenge. Being Overweight or obese is not only a growing problem among children and adolescents,^[2]^ and the situation is dire among the adult population.^[3]^ By 2025, study predict that the global obesity rate will reach 18% for men and over 21% for women.^[4]^ At the same time, overweight and obesity are long-established areas of research, most studies have shown that lifestyles, such as the amount of alcohol consumed, activity or exercise, and changes in socio-economic production patterns are the main reasons for the overall increase in obesity rates over the years. Age, gender, ethnicity, place of residence, geographical location, marital status, education, smoking or alcohol consumption, and family history of cardiovascular disease are all associated with the risk of developing overweight or obesity at this stage.^[5]^

In general, being overweight or obese can cause a range of physical and psychological problems, such as the risk of cardiovascular disease, non-communicable diseases such as immune-endocrine system disorders, and an increased risk of infection from the new coronary pneumonia in today pandemic.^[6–9]^ Obese children are more likely to experience breathing difficulties, increased risk of fractures, hypertension, early signs of cardiovascular disease, insulin resistance, and psychological effects.^[10–12]^ The complications associated with obesity not only reduce the quality of life of individuals but also overwhelm the national healthcare system as well as being a serious economic burden.^[13]^ In this regard, the intervention and treatment of obesity require a combination of lifestyle modification, psychological interventions, and pharmacological and surgical therapies. Research has shown that in the long term, psycho-cognitive interventions can be more effective when incorporated into a comprehensive intervention.^[14]^ Episodic Future Thinking (EFT), as a form of psycho-cognitive therapy, typically refers to the process of guiding participants to mentally pre-experience future events beyond the present, that is, by imagining and describing positive, personal future events, thereby prompting the individual to place greater future rewards over smaller immediate rewards.^[15,16]^ Linking future goal events to current events in as much detail as possible to help develop cues they can recall on a daily basis promotes health-related decision-making (For example, “Taking a family holiday at the end of this month and people are complimenting me on my glowing skin and healthy body”）.^[17]^ This ability can often be acquired through training, and EFT-based interventions are associated with positive health outcomes.^[18]^

EFT-based interventions for people with overweight or obesity have been used by researchers as part of comprehensive cognitive training or on their own,^[19]^ however, there is limited discussion of the specific application and effectiveness of such interventions in specific scenarios in people with overweight or obesity, and appropriate tools must be used effectively to enhance their effectiveness. Therefore, we undertook the current scoping review to synthesize and describe recent EFT in people with overweight or obesity. Therefore, we conducted the current scoping review to synthesize and describe recent EFT in people with overweight or obesity. By conducting a scoping review of the relevant literature, we were able to compare clinical endpoint outcomes and identify scenario-specific applications of EFT in people with overweight or obesity to inform efforts to address the obesity epidemic.

## 2. Methods

This scoping review was conducted according to the Preferred Reporting Items for Systematic Reviews and Meta-Analyses—Extension for Scoping Reviews (PRISMA-ScR)^[20]^ (checklist: Supplementary Table 1, http://links.lww.com/MD/J245). The review protocol was not prospectively registered. As the intent of this review was to scope the breadth of research, rather than reach specific conclusions, we chose not to undertake the optional risk of bias assessment.^[21]^

### 2.1. Research questions

This study uses the conceptual framework proposed by Arksey and O’Malley.^[22]^ The following steps were followed: identifying the research question, identifying relevant studies to answer those research questions, selecting of study, charting of data, and reporting and summarizing the result. An initial review of the relevant literature and discussions with team members were identified. The research questions included in this scoping review are as follows:(which are also shown in the attached Supplementary Table 2 http://links.lww.com/MD/J246):

RQ1: What are the prerequisites for the application of EFT in people with overweight or obesity?

RQ2: What are the modalities and components of EFT interventions in people with overweight or obesity?

RQ3: What are the key outcome indicators of EFT interventions applied in people with overweight or obesity?

RQ4: What are the outcomes of EFT interventions in people with overweight or obesity?

### 2.2. Inclusion and exclusion criteria

Inclusion criteria: English language literature; the study was conducted on a population in need of weight control and the data could be extracted in full; the study was on the current status and effectiveness of the application of EFT to a population in need of weight control; the type of literature was a quantitative study or a quantitative part of a mixed study. Exclusion criteria: literature types such as reviews, systematic evaluations, qualitative studies, and conference abstracts; literature for which the full text is not available; duplicate publications.

### 2.3. Search strategies

**Figure 1.**
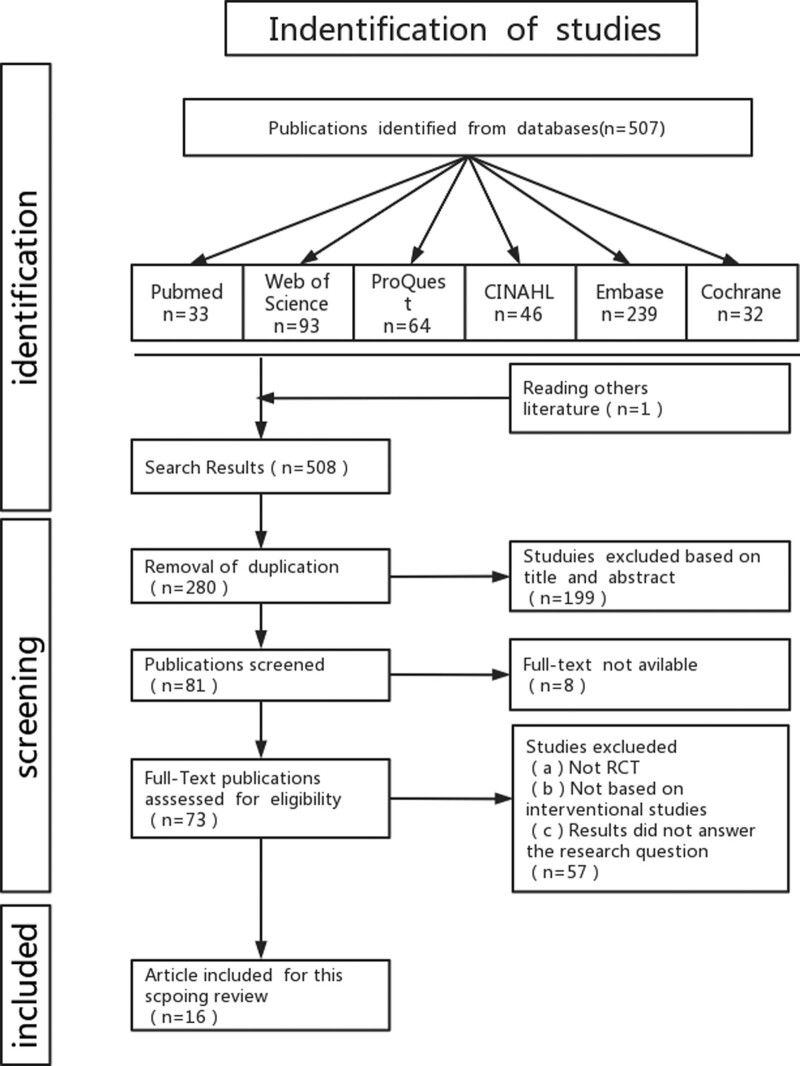
Flow diagram.

A combination of subject terms and free terms were used for the study and a total of 6 databases were used: PubMed, Web of Science, ProQuest, CINAHL, Embase, and Cochrane. The searches were performed on December 28, 2022. Therefore, studies published before December 28, 2022, were included in this review. The English search terms were: “(1) (Episodic Future thinking); (2) (obesity) OR (obese) OR (overweight) OR (over-weight) OR (Body Weight) OR (Obesity, Abdominal) OR (Body Weight) OR (losing weight); (3) (eating) OR (food) OR (food intake) OR (Intake, food) OR (Snacks) OR (Snack food) OR (food, Snack) OR (foods, Snacks) OR (snack foods) OR (snack time) OR (snack times) OR (snacking). They are connected by a Boolean search formula: “#1AND (#2OR#3) ”(The specific search strategy is shown in the attached Supplementary Table 3, http://links.lww.com/MD/J247). The researcher backdated the relevant references of the included literature to improve the rate of completeness, as shown in Figure 1.

### 2.4. Identification of literature and data extraction

The retrieved literature was imported into NoteExpress software and duplicates were removed. Two researchers conducted an initial screening of the reading titles and abstracts according to the literature inclusion criteria, and then read the full text for the 3rd screening to identify the included literature, and in case of disagreement, discussion or the 3rd researcher assisted in the judgment. Information was extracted by entering information about the literature according to a pre-designed form, mainly including the authors, country, year of publication, type of study, intervention/study population, sample size, evaluation indicators, conclusions, etc.

## 3. Results

### 3.1. Overall descriptive results

Of the 16 manuscripts included, all were randomized controlled trials and met the NIH definition of a clinical trial.^[23]^

In the analysis used to extract information from the studies, the results are summarized in Table 1.

**Table 1:** Basic characteristics of the literature for inclusion in this analysis (n = 16).

Author, yr (country)	Study design	Participants	Duration/mode of intervention	Intervention	Control group	Result indicators
Epstein H et al2022 (United States of America) ^[24]^	RCT	64 participants with overweight or obesity	6-mo/MAMRT app	EFT + BWL	DCI + BWL	DD; weight; HbA1c; physical activity
Athamneh et al (2021 (United States of America) ^[25]^	RCT-2 × 2 factorial design	255 participants with obesity	24 h/Computer (Laboratory studies)	EFT-health goal; EFT-general	ERT-health goal; ERT-general	DD; craving fast food
Mansouri et al 2021 (United States of America) ^[26]^	RCT	33 participants with overweight or obesity	1-wk/MAMRT app	EFT	ERT	DD; energy intake; RRV of food
Segovia et al 2020 (United States of America)^[27]^	RCT-3 × 3 factorial design	94 participants with overweight, and 80 participants with obesity	Immediate intervention/Face-to-face experiments	EFT	Health information condition; Blank control	Food choice; BMI;
Leahey et al 2020(United States of America)^[28]^	RCT (Ongoing)	Participants with overweight or obesity	4-mo/Online and offline integration	WLM + EFT	WLM − STD	DD; physical activity; weight
Stein et al 2020 (United States of America)^[29]^	RCT	78 participants with overweight or obesity	Immediate intervention/Face-to-face experiments	EFT	ERT	DD; Food purchase task
Levens et al 2019 (United States of America)^[30]^	RCT-2 × 2 factorial design (Ongoing)	156 participants with overweight or obesity	5 d/Qualtrics survey software	PosA-EFT; neutral affect + EFT	PosA + no EFT; neutral affect+ERT	TD; Food Demand; Food Choice
Hollis-Hansen et al 2019 (United States of America)^[31]^	RCT	Study1:24 participants with overweight or obesity; Study2:33 participants with overweight or obesity;	Immediate intervention/Face-to-face experiments	Study 1: EFT Study 2: EFT_GDP_	Study 1: savings; Study 2: EFT_GEN;_ ERT	calories/person; calories per- chased
Kakoschke et al 2018 (Australia)^[32]^	RCT	60 participants with overweight or obesity	6-wk/smartphone	AAT, EFT	Blank control	Food choice; Weight;DD
Sze et al 2017 (United States of America)^[33]^	Study1: RCT; Study2: RCT-2 × 3 factorial design	Study1: 66 participants with overweight or obesity Study2: 204 participants with overweight or obesity	Immediate intervention/Face-to-face experiments	Study1: Online EFT Study2: EFT; negative income shock	Study1: ERT Study2: neutral income narrative; ERT; NoET	Study1: DD; BMI; Study2: DD; Food demand
Stein et al 2017 (United States of America)^[34]^	RCT	131 participants with overweight or obesity	Immediate intervention/Qualtrics online survey software	EFT-1; EFT-3	ERT-1; ERT-3	DD; BMI
O’Neill et al 2016 (United States of America)^[35]^	RCT	29 women with overweight or obesity	1 d/smartphone	EFT	ERT	Weight and height; Calorie and macronutrient intake
Sze et al 2015 (United States of America)^[36]^	RCT	20 parent-child dyads with overweight or obesity	4-wk/MAMRT	EFT	NIT	BMI; percent overweight; energy Intake;
Daniel et al 2015 (United States of America)^[37]^	RCT	42 kids with overweight or obesity (9–14-yr-old)	Immediate intervention/Face-to-face experiments	EFT	ERT	DD; restraint; Weight;
Daniel et al 2013 (United States of America)^[38]^	RCT	24 women with overweight or obesity	two 90 min sessions, 3–7 d apart/Face-to-face experiments	EFT	CET	DD; Weight; Time perspective; BIS/BAS
Daniel et al 2013 (United States of America)^[39]^	RCT	26 Women with overweight or obesity	Immediate intervention/Face-to-face experiments	EFT	ERT	DD; calories; degree of imagery

Abbreviations: AAT = approach-avoidance training, BIS/BAS = behavioral inhibition/behavioral approach scale, BWL = behavioral weight loss, CET = control episodic thinking, DCI = Check In Daily, EFT = episodic future thinking, EFT_GDP_ = Goal-directed process EFT, EFT_GEN_ = general EFT, ERT = episodic recent thinking, DD = delay discounting, MAMRT app = mobile audio manager and response tracker app, NIT = nutrition information thinking, NoET = no episodic thinking, PosA = positive affect, RRV = high relative reinforcing value, TD = temporal discounting, WLM − STD = weight loss maintenance program.

Depending on the type of study taken in the selected studies, this was as follows: 12 of the studies took a Parallel-RCT^[24–35]^ and the other 4 took a 2 × 2 or 2 × 3 factorial RCT.^[33,36,38,39]^

Further requirements for inclusion of overweight/obese patients, that is, the presence of motivation or willingness to lose weight, were made in 3 of the 16 articles and the importance of this was highlighted in the articles.^[30,31,39]^

Regarding the form of the intervention, 8 of the interventions were conducted using the internet or smartphone reminders based on a specific app, and the outcome was measured through a delayed discounting task after a period of intervention in daily life or conjunction with a weight-loss program.^[24–26,29,31,32,34,38]^ The other 8 interventions were conducted in a face-to-face setting, with a laboratory study followed by a delayed discounting task.^[27,28,30,33,35–37,39]^

Regarding the post-implementation evaluation of the intervention, more than half of the articles (62.5%) conducted a post-implementation evaluation of the effectiveness and satisfaction with the use and development of the measure^[27,28,30,32–37,39]^ and were positive about the effectiveness of the EFT intervention in the people with overweight or obesity.

### 3.2. Specific synthesized results

Daniel et al^[35]^ showed in a laboratory study that EFT participants consumed fewer calories than control-EFT participants, constituting the first evidence that EFT led women with overweight or obesity tempted by the immediate gratification of unhealthy foods to reduce their delay discounting (DD) and energy intake, an important self-regulatory skill in maintaining a healthy weight.

Epstein H et al^[24]^ conducted a controlled study of obese patients with diabetes in the maintenance phase of weight loss who were on a weight loss program while incorporating an EFT intervention, using the Mobile Audio Manager and Response Tracker app that provided the opportunity to have EFT cues provided to participants on their smartphones and answer questions about what decisions they were using cues to assist them. opportunity to have EFT cues provided to participants on their smartphones and answer questions about what decisions they were using cues to assist them in making. Leahey et al^[26]^ also conducted an EFT intervention in an overweight/obese population during the weight loss maintenance phase to examine whether EFT improves DD within the context of weight loss maintenance. The study not only demonstrated the effectiveness of the remote intervention but also the relevance of applying EFT as a cognitive behavioral intervention specifically to the daily lives of people with overweight or obesity.

Mansouri et al^[25]^ explored the effects of daily EFT on delayed discounting, energy intake, and the effect of the relative reinforcement value of food. Compared to ERT (Episodic Recent Thinking) controls, it was ultimately found that repeated EFT did not reduce DD, energy intake, or RRV of food, which is different from previous studies that have yielded results. Kakoschke et al^[29]^ compared EFT with another cognitive training approach-avoidance training (AAT) which intervention focused on changing individual appetite cues for unhealthy foods and then measured food choice and weight change. Results showed that healthy food choices were higher in the AAT group than in the control group and that weight loss from pre-training to 6 weeks follow-up was observed in the AAT group, but not in the EFT or control groups. Contrary to previous findings, EFT does not affect delayed discounts or food choices.

Previous studies of EFT involving eating habits typically include -- low vs high-calorie food choices, time intervals between food intake, and amount of food intake.^[25,27,29,37,38]^ Segovia et al^[37]^ investigated how EFT influenced food choices in people with normal weight, overweight, or obesity through a laboratory study. A before-and-after control by itself concluded that EFT has a positive impact on the food choices of people with obesity only. Meanwhile, it explained that the difference in self-perception makes the application of EFT ineffective in people with overweight or obesity.

Levens et al^[38]^ introduced positive emotions (PosA) associated with healthy food choices into dietary choices through a 2 × 2, randomized, laboratory-based intervention study in people with overweight or obesity. A brief guided imagery exercise was conducted to address abstinence (EFT: yes, no) and reward (PosA imagery: positive, neutral) mechanisms of eating behavior, with delayed discounting and food intake as the primary outcomes measured. The program was designed to test the independent and interactive effects of EFT and PosA on Temporal Discounting, food choice, and food demand in adults with overweight or obesity, and further advancement is still ongoing. A follow-up study introduced the role of health goals in EFT interventions by establishing 2 control conditions in Study 2: ERT-Health Goals, which included health goals, and ERT-General. The effects of health goals and general EFT on DD in an obese population were examined, as well as fast food needs and cravings in obese individuals. Results showed that expanding future thinking by including health goals may promote healthy decision-making and lead to positive behavior change.^[36]^

One study in this scoping review^[39]^ proposed that study participants need to be motivated or willing to lose weight, this study was divided into 2 experimental groups and recruited only participants interested in weight loss and excluded people with depression, as people with depression can impair positive thoughts and experiences and therefore the usefulness of EFT as a cognitive intervention, an exclusion criterion also reflected in related studies.^[30]^ In the second set of experiments, the effect of EFT on DD and food needs were explored concerning income shocks (i.e., sudden shifts to poverty). In both sets of experiments, EFT administered online was found to reliably reduce DD. Even in the face of negative income shocks, EFT reduced DD and the demand for fast food. This is consistent with the study by Stein et al.^[27]^

Stein et al^[30]^ validated a 5-trial adjustment delay task in an obese population to accommodate frequent and rapid measures of DD. while adjusting for the number of future events generated and imagined by participants. Ultimately, it was found that the reduction in DD may depend on the future imagined in multiple time frames.

O’Neill et al^[31]^ conducted a controlled experiment of EFT versus ERT in 29 participants with overweight or obesity to validate the use of EFT in a natural setting, showing that the EFT group consumed significantly fewer calories (540.44 ± 178.20 Mean ± SD) than the ERT group (749.32 ± 169.90; *F*(1, 27) = 10.38, *P* = .003). EFT may be an important adjunct to traditional behavioral therapy for changing the diet of habitual binge eaters.

Because of the particularity of delayed gratification level in children with overweight or obesity, the researchers verified the effectiveness of EFT in the treatment of childhood obesity through the intervention of EFT in children.^[33]^ Through the free eating task and online DD task, the calorie intake and DD level of obese children under the intervention of EFT were measured. The results showed that Overweight/obese children showed less DD in the EFT condition and ate less than the control group in the near-term thinking condition. The conclusion is that EFT could be a useful tool for overweight/obese children attempting to control their eating for weight loss. On this basis, a follow-up study was carried out on children and family EFT intervention in weight management. EFT was applied to an intervention for obesity treatment in the parent-child group,^[32]^ and in a natural diet setting, the control group was provided with only nutritional information training and intervention via a mobile device, and individualized nutritional cues were generated, focusing on the recording of positive future events. family-based EFT interventions improve eating behaviors in adults and children. Hollis-Hansen et al^[28]^ applied EFT to overweight/obese mothers and major home shoppers. Through a controlled experiment, they mainly compared the calories of food purchased by home shoppers. This study also set strict consumption standards for participants, and the final result showed that EFT could reduce the purchase of food with high calories. Ultimately, family-based dietary interventions can be implemented to improve weight management. EFT has been shown in the laboratory to reduce dietary calorie intake in overweight/obese women.^[34]^

## 4. Discussion

### 4.1. People with overweight or obesity and DD

DD describes the tendency to prefer smaller, short-term rewards over larger, long-term rewards. The overvaluation of instant rewards and the discounting of future rewards, also known as high DD, is an important factor in affecting how people make decisions.^[40]^ Compared to individuals of healthy weight, people with overweight or obesity have higher DD, usually reflected in low self-control and high impulsivity, and have a greater tendency to choose instant rewards, even when well-fed. Also manifested in diet or activity management, they tend to choose to consume foods which energy-dense, high in salt, sugar, and saturated fat or even overeat, etc., choosing rest over exercise, and neglecting long-term health, which further leads to weight gain.^[41,42]^ EFT as a form of cognitive training can help people with overweight or obesity understand the phenomenon of DD in health behavior management and shift their gaze from the “immediate” to the “future” when faced with decisions related to health behaviors decision. This reduces the impulsivity of participants, and help to choose behaviors that are beneficial for long-term health (e.g., choosing low-oil foods over high-oil foods, exercise over rest, etc), and studies have shown that EFT is more effective in diet management.^[43–45]^ Children or women with overweight or obesity exhibit higher DD and place a higher subjective value on the instant consumption of attractive, fattening foods than on future long-term health goals. Existing research has also incorporated the effectiveness of EFT interventions for such populations with high DD levels and the ability to delay gratification as a promising goal for cognitive-behavioral interventions.^[46,47]^

### 4.2. Conditions for the Use of EFT in people with overweight or obesity

Current research has shown that most studies take into account the psychological status of the individual and their willingness to lose weight when including a study population. Studies that require the use of mobile devices or computer-assisted interventions also need to consider individual compliance with the intervention, including but not limited to complications (such as carpal tunnel syndrome) when the individual uses the device. Research has shown that individuals with depression tend to show negative perceptions of future thinking,^[48]^ which can lead to poor assessments of future possibilities or inappropriate processing of future-related cues, compromising the effectiveness of EFT as a cognitive intervention. Existing studies have used the Patient Health Questionnaire-9 as an exclusion criterion when screening samples in an attempt to reduce bias.^[30]^ However, this screening is limited in the available intervention studies, which must take into account that people with overweight or obesity are more likely to exhibit anxiety or depressive tendencies,^[49]^ or even to experience emotional eating, further exacerbating obesity, which is more pronounced in adolescent individuals and obese women but mostly may not reach depression in the clinical sense.^[50]^ At the same time, an individual preexisting motivation, which includes both self-coordinated (want to achieve) and non-self-coordinated (must achieve) goals, is important for the success of EFT interventions. In general, when individuals exhibit self-coordinated goals prior to EFT interventions, that is, those who are more impulsive but willing to take the initiative to achieve health-related goals, such individuals are more effective than those who do not express a desire to change their behavior. They are more likely to associate their health decisions with future time cues, such as “I will wear a beautiful dress to a party in 2 months,” and have a stronger sense of “reality” and experience the future in advance, making EFT interventions more sustainable and effective. This makes EFT interventions more sustainable and effective.^[51,52]^ However, these conditions do not mean that EFT is a limited intervention for people with overweight or obesity. EFT can be used as part of or in addition to cognitive-behavioral therapy for people with overweight or obesity. It is possible that tailoring behavioral obesity treatment by incorporating EFT training for people with overweight or obesity may improve weight loss success. Therefore, EFT is not exclusive.

### 4.3. Applied in people with overweight or obesity as a form of intervention

On the EFT intervention, Participants were encouraged to think about events for which being active, fit, or at a reduced weight would be particularly salient (e.g., dancing at a nephew wedding in a favorite dress), and when it came to health-related choices (e.g., rest OR exercise, whether to consume foods high in oil) triggered the cue, prompting participants to make a new choice.

When applied to people with overweight or obesity, EFT interventions take many forms, including mobile device or internet-based, face-to-face interventions, and a combination of online and offline interventions. App or platforms are similar to Mobile Audio Manager and Response Tracker have been used to create, prompt and manage cues (voice or image) for EFT in people with overweight or obesity, and have proven in practice the feasibility of web-based interventions for health management in today “internet plus” trend.^[25,32]^ EFT can be effectively administered remotely, specifically online.^[47]^ It is simple, convenient, and more adapted to the contemporary food intakes environment, such as the development and convenience of food retailing conditions,^[53]^ and the mobile devices that facilitate the process of EFT interventions for people with overweight or obesity, including the regulation of diet and health-related decisions. At the same time, the transition from a laboratory intervention to an intervention in a natural environment was achieved. The researchers ensured that participants completed the EFT intervention during natural eating as much as possible, limiting the impact of participant demand expectations and reducing bias.^[31]^ EFT proved to be implementable even in a very distracting environment. Future studies should also focus on a lived-in experimental setting.

When applying EFT to people with overweight or obesity, the content of the EFT intervention, the intervention node, and the number of interventions can all impact the intervention effectiveness. In this scoping review, the effects of perspective in EFT were overwhelmingly isolated by controlling with ERT (i.e., in which participants imagined real events that had recently occurred), by ensuring that situational thinking in both the experimental and control groups involved memory, personal details, and were matched to vividness. In terms of intervention content, the vividness, specificity, Episodicity, Future-Orientedness, and Content Specificity of EFT need to be taken into account as Core Components of EFT. The effect of EFT on DD-related behavior is more pronounced when the vividness or specificity of future simulated events increases.^[52]^ Often participants can create single or even multiple cues, which can be updated at different stages, and can be adjusted or updated based on feedback each time they decide on physical activity and diet, the content of these cues is not static.^[24]^ The cues can also be presented in a variety of formats, including a combination of text, audio, images, etc. Whatever the format, participants are asked to describe the cues as vividly as possible (e.g., in 6-month time I will be dancing on the beach in a beautiful dress), thus increasing the recall effect after each cue.^[52]^ At the same time, expanding EFT by incorporating health goals may promote healthy decision-making and lead to positive behavior change. Research has shown that the inclusion of health goals in general EFT has a significantly higher impact on the intensity and elasticity of fast-food demand.^[36]^ The application of single EFT versus repeated multiple EFT interventions in people with overweight or obesity also needs to be further discussed, with studies suggesting that repeated exposure to EFT does not reduce DD, energy intake, or RRV of food.^[25]^

### 4.4. Outcomes of EFT intervention in people with overweight or obesity

More rigorous research is needed to ascertain the effectiveness of manipulating time perspective in improving eating behaviors before such interventions are more widely used.

EFT is used in interventions for people with overweight or obesity and the main outcome indicators include DD (81.25%), weight/BMI (56.25%), food/energy intake/choice (50%), and physical activity (12.5%). Previous studies have identified DD as one of the targets for the treatment of obesity and as one of the main outcome indicators for cognitive behavioral interventions. Existing research has demonstrated that EFT can extend the temporal scope of intertemporal choice,^[54]^ and reduce DD by vividly simulating and imagining the future. In a follow-up study, the effect of DD was influenced by the efficacy of the imagined event, whether positive imagery reduced DD, and whether negative imagery increased DD.^[38]^ Although most of the current results on EFT interventions for people with overweight or obesity show some reduction in DD in the EFT group compared to the pre-experimental period, with a consequent reduction in weight or food intake, there are still studies that have reached different conclusions than before.^[24]^

We thus found that the following issues influenced the conclusions, the timing of DD measurement, the sequential order of the experiments, and the baseline survey of participants; most of the measurements of DD in this review used the monetary delay-discounting task, although previous studies have concluded that studies using monetary reward experiments generally report no association with those using food Although previous studies have concluded that studies using monetary reward experiments generally report no association, with a clear positive association in studies using food reward experiments, there is no uniform conclusion that the measurement approach applied in this review is a “best practice.”^[52]^ However, the timing of the measurement of DD still needs to be discussed, with outcome measures (DD and food/energy intake/choice) being taken after the completion of EFT training, the effect of EFT on these measures is likely to be strongest at (or close to) the time when participants imagine their EFT cues, and thus some studies suggest that EFT should be administered immediately before outcome assessment, but again some studies have demonstrated that the intervention effects of EFT are somewhat delayed and can maintain their utility to some extent through intervention training, which may be related to the vividness of the cue and the number of subsequent cue cues.

Most of the previous studies have been conducted in the laboratory, and although subsequent studies have attempted to conduct them in a natural setting, they have mostly focused on single management such as diet management and weight management in people with overweight or obesity, and have rarely been applied in a truly integrated manner to actual weight maintenance. In the combined application, there may be a ceiling effect due to the intensive and effective weight loss program undertaken upfront, which in turn may make the EFT measurements insignificant. However, this does not mean that EFT is meaningless as an adjunct to weight management, as it is expected to play an integrative rather than additive role in interventions for people with overweight or obesity. At the same time, future studies will often re-screen participants at baseline to ensure that the intervention is scientifically sound and relevant, including the ability to establish future scenario cues and adherence, thus ensuring the successful conduct of the intervention.

## 5. Limitations and future directions

There were several limitations to the literature included in the scoping review. First, many of the included studies were small sample sizes, with only 16 intervention studies (two of which are ongoing), and the scoping review was limited to controlled studies of EFT applied in people with overweight or obesity, with no reporting on other specific weight management interventions in the literature. Second, the participants were disproportionately white, and intervention studies were mostly conducted in North America and Australia but not elsewhere. The EFT mentioned in this review may show cultural variability across geographic regions,^[55]^ and future studies in a broader population are necessary to replicate the current results. Also, the application of EFT to specific types of overweight or obesity was not discussed in this scoping review, and it is important to emphasize that the management of people with overweight or obesity and interventions need to consider a combination of measures, such as simple obesity, stress obesity, pathological obesity or obesity resulting from the course of disease treatment, to achieve interventions in weight management behaviors from psycho-physical-social aspects when necessary.^[56,57]^ EFT as a psychological intervention that intervenes in the individual perception of time, which leads to behavioral change and ultimately focuses on weight management behaviors that are not absolutely effective and consistent across overweight or obesity types. Future studies could also be conducted on multiple overweight or obese types, such as the breast cancer patients with overweight or obesity in the Sukumar study, where compliance with EFT interventions and changes in weight and DD rates were documented by comparing them with ERT.^[58]^ In addition, the findings reported in this review are a broad description of current specific measures of EFT applied to people with overweight or obesity and do not describe specific strategies that are limited, such as the best content or frequency of situational cues for people with overweight or obesity, and future studies will need to fit the cues and frequency of content that are most appropriate for the individual in practice and describe the results.

## 6. Conclusions

The overall conclusion of this review is that although there are certain prerequisites for the application of EFT to weight management in people with overweight or obesity, EFT is a highly portable and effective technique for reducing intake in complex dietary settings. Through the development and training of online management devices, individuals who have learned EFT and use situational cues to make individuals more future-oriented in their daily decision-making can direct their cognitive focus towards long-term goals rather than the short-term pleasures associated with the common variety of tempting foods. EFT may be an important adjunct to traditional behavioral therapies and could easily be used as a potential treatment tool in clinical and outpatient rehabilitation programs for people with overweight or obesity.

## Acknowledgments

Thanks to all those who helped with this article.

## Author contributions

**Conceptualization:** Yuchen Liu, Sufang Huang, Danni Feng, Xiaorong Lang.

**Formal analysis:** Yuchen Liu, Danni Feng.

**Methodology:** Yuchen Liu.

**Validation:** Yuchen Liu.

**Writing – original draft:** Yuchen Liu.

**Writing – review & editing:** Yuchen Liu, Sufang Huang, Danni Feng, Xiaorong Lang, Quan Wang, Kexin Zhang.

## Supplementary Material






