# Supplementary material for: The use of episodic future thinking in people with overweight or obesity: A scoping review
Source: Medicine (Baltimore). 2023 Jul 28;102(30):e34269. doi: 10.1097/MD.0000000000034269 (PMC10378810; doi:10.1097/MD.0000000000034269)
Supplement: Supplementary file 2 [file medi-102-e34269-s002.pdf]

Supplementary Table 2-Research question

	Question
RQ1:	What are the prerequisites for the application of EFT in people with overweight or obesity?
RQ2:	What are the modalities and components of EFT interventions in people with overweight or obesity?
RQ3:	What are the key outcome indicators of EFT interventions applied in people with overweight or obesity?
RQ4:	What are the outcomes of EFT interventions in people with overweight or obesity?
