# Supplementary material for: The use of episodic future thinking in people with overweight or obesity: A scoping review
Source: Medicine (Baltimore). 2023 Jul 28;102(30):e34269. doi: 10.1097/MD.0000000000034269 (PMC10378810; doi:10.1097/MD.0000000000034269)
Supplement: Supplementary file 3 [file medi-102-e34269-s003.pdf]

Supplementary Table 2 – Search strategies and number of results obtained across the six searched databases

PubMed (1960+)

#	Search Syntax	28/12/2022
1	"episodic future thinking"[Title/Abstract]	273
2	"Obesity"[MeSH Terms] OR "Overweight"[MeSH Terms]	263,957
3	"obesity"[Title/Abstract] OR "obese"[Title/Abstract] OR "overweight"[Title/Abstract] OR "over-weight"[Title/Abstract] OR "body weight"[Title/Abstract] OR ("obesity, abdominal"[MeSH Terms] OR ("obesity"[All Fields] AND "abdominal"[All Fields]) OR "abdominal obesity"[All Fields] OR ("obesity"[All Fields] AND "abdominal"[All Fields]) OR "obesity abdominal"[All Fields]) OR "body weight"[Title/Abstract] OR "losing weight"[Title/Abstract]	685,348
4	"eating"[Title/Abstract] OR "food"[Title/Abstract] OR "food intake"[Title/Abstract] OR "intake food"[Title/Abstract] OR "Snacks"[Title/Abstract] OR "snack food"[Title/Abstract] OR "food snack"[Title/Abstract] OR "foods snacks"[Title/Abstract] OR "snack foods"[Title/Abstract] OR "snack time"[Title/Abstract] OR "snack times"[Title/Abstract] OR "snacking"[Title/Abstract]	594,870
5	“#1AND（#2OR#3OR#4）”	33

Web of Science

#	Search Syntax	28/12/2022
1	((TS=(episodic future thinking)) OR TI=(episodic future thinking)) OR AB=(episodic future thinking)	1,322

2	((TS=((obesity) OR (obese) OR (overweight) OR (over-weight) OR (Body Weight)OR (Obesity, Abdominal) OR (Body Weight) OR (losing weight))) OR TI=((obesity) OR (obese) OR (overweight) OR (over-weight) OR (Body Weight)OR (Obesity, Abdominal) OR (Body Weight) OR (losing weight))) OR AB=((obesity) OR (obese) OR (overweight) OR (over-weight) OR (Body Weight)OR (Obesity, Abdominal) OR (Body Weight) OR (losing weight))	1,876,075
3	((TS=((eating) or (food) or (food intake) or (Intake, food) or (Snacks) or (Snack food) or (food, Snack) or (foods, Snacks) or (snack foods) or (snack time) or (snack times) or (snacking))) OR TI=((eating) or (food) or (food intake) or (Intake, food) or (Snacks) or (Snack food) or (food, Snack) or (foods, Snacks) or (snack foods) or (snack time) or (snack times) or (snacking))) OR AB=((eating) or (food) or (food intake) or (Intake, food) or (Snacks) or (Snack food) or (food, Snack) or (foods, Snacks) or (snack foods) or (snack time) or (snack times) or (snacking))	3,943,914
4	“#1AND（#2OR#3）”	93

ProQuest<br>＃	Search Syntax	28/12/2022
1	title((Episodic Future thinking)) OR summary((Episodic Future thinking)) OR abstract((Episodic Future thinking)) OR subject((Episodic Future thinking))	981
3	title((obesity) OR (obese) OR (overweight) OR (over-weight) OR (Body Weight)OR (Obesity, Abdominal) OR (Body Weight) OR (losing weight)) OR summary((obesity) OR (obese) OR (overweight) OR (over-weight) OR (Body Weight)OR (Obesity, Abdominal) OR (Body Weight) OR (losing weight)) OR abstract((obesity) OR (obese) OR (overweight) OR (over-weight) OR (Body Weight)OR (Obesity, Abdominal) OR (Body Weight) OR (losing weight)) OR	1,783,333

	subject((obesity) OR (obese) OR (overweight) OR (over-weight) OR (Body Weight)OR (Obesity, Abdominal) OR (Body Weight) OR (losing weight))	
4	title((eating) OR (food) OR (food intake) OR (Intake, food) OR (Snacks) OR (Snack food) OR (food, Snack) OR (foods, Snacks) OR (snack foods) OR (snack time) OR (snack times) OR (snacking)) OR summary((eating) OR (food) OR (food intake) OR (Intake, food) OR (Snacks) OR (Snack food) OR (food, Snack) OR (foods, Snacks) OR (snack foods) OR (snack time) OR (snack times) OR (snacking)) OR abstract((eating) OR (food) OR (food intake) OR (Intake, food) OR (Snacks) OR (Snack food) OR (food, Snack) OR (foods, Snacks) OR (snack foods) OR (snack time) OR (snack times) OR (snacking)) OR subject((eating) OR (food) OR (food intake) OR (Intake, food) OR (Snacks) OR (Snack food) OR (food, Snack) OR (foods, Snacks) OR (snack foods) OR (snack time) OR (snack times) OR (snacking))	9,028,799
4	“#1AND（#2OR#3）”	64

CINAHL #	Search Syntax	28/12/2022
1	TI episodic future thinking OR SU episodic future thinking OR AB episodic future thinking	978
2	TI ((obesity) OR (obese) OR (overweight) OR (over-weight) OR (Body Weight)OR (Obesity, Abdominal) OR (Body Weight) OR (losing weight)) OR SU ((obesity) OR (obese) OR (overweight) OR (over-weight) OR (Body Weight)OR (Obesity, Abdominal) OR (Body Weight) OR (losing weight)) OR AB ((obesity) OR (obese) OR (overweight) OR (over-weight) OR (Body Weight)OR (Obesity, Abdominal) OR (Body Weight) OR (losing weight))	1,899,336
3	TI ((eating) OR (food) OR (food intake) OR (Intake, food) OR (Snacks) OR (Snack food) OR (food, Snack) OR (foods, Snacks) OR (snack foods) OR (snack time)	5,158,549

	OR (snack times) OR (snacking)) OR SU ((eating) OR (food) OR (food intake) OR (Intake, food) OR (Snacks) OR (Snack food) OR (food, Snack) OR (foods, Snacks) OR (snack foods) OR (snack time) OR (snack times) OR (snacking)) OR AB ((eating) OR (food) OR (food intake) OR (Intake, food) OR (Snacks) OR (Snack food) OR (food, Snack) OR (foods, Snacks) OR (snack foods) OR (snack time) OR (snack times) OR (snacking))	
4	“#1AND（#2OR#3）”	46

Embase (OVID interface, 1860+)

#	Search Syntax	28/12/2022
1	Episodic Future thinking. mp. [mp=ti, ab, tx, ct, sh, hw, tn, ot, dm, mf, dv, kf, fx, dq, bt, nm, ox, px, rx, an, ui, ds, on, sy, ux, mx]	982
2	(obesity or obese or overweight or over-weight or Body Weight or Obesity, Abdominal or Body Weight or losing weight).mp. [mp=ti, ab, tx, ct, sh, hw, tn, ot, dm, mf, dv, kf, fx, dq, bt, nm, ox, px, rx, an, ui, ds, on, sy, ux, mx]	4,142,067
3	(eating or food or food intake or Intake, food or Snacks or Snack food or food, Snack or foods, Snacks or snack time or snack times or snacking).mp. [mp=ti, ab, tx, ct, sh, hw, tn, ot, dm, mf, dv, kf, fx, dq, bt, nm, ox, px, rx, an, ui, ds, on, sy, ux, mx]	3,043,427
4	“#1AND（#2OR#3）”	238

Cochrane #	Search Syntax	28/12/2022
1	(obesity) OR (obese) OR (overweight) OR (over-weight) OR (Body Weight)OR (Obesity, Abdominal) OR (Body Weight) OR (losing weight) OR (eating) OR (food) OR (food intake) OR (Intake, food) OR (Snacks) OR (Snack food) OR (food, Snack) OR (foods, Snacks) OR (snack foods) OR (snack time) OR (snack times) OR (snacking) in Title Abstract Keyword AND Episodic Future thinking in Title Abstract Keyword	32
